# Exploring Electrochemical Methods for Precision Stress Control in Nanoscale Devices

**DOI:** 10.1021/acs.nanolett.5c02870

**Published:** 2025-08-13

**Authors:** Di Chen, Natasa Vasiljevic, Andrei Sarua, Martin Kuball, Krishna C. Balram

**Affiliations:** † School of Physics, H.H. Wills Physics Laboratory, 1980 University of Bristol, Bristol BS8 1TL, United Kingdom; ‡ Centre for Device Thermography and Reliability, School of Physics, H.H. Wills Physics Laboratory, University of Bristol, Bristol BS8 1TL, United Kingdom; § Quantum Engineering Technology Laboratories and School of Electrical, Electronic and Mechanical Engineering, University of Bristol, Bristol BS8 1UB, United Kingdom

**Keywords:** electrochemistry, stress tuning, nanoscale devices, palladium−hydrogen absorption, Raman shift, stress−strain

## Abstract

Tuning the local film stress (and associated strain) provides a universal route toward exerting dynamic control on propagating fields in nanoscale geometries and engineering controlled interactions between them. The majority of existing techniques are adapted for engineering either uniform stresses or fixed stress gradients, but there is a need to develop methods that can provide independent precision control over the local stress at the nanoscale in the 2D plane. Here, we explore electrochemical absorption of hydrogen in structured palladium thin-film electrodes and the associated shape-dependent stress to engineer controlled, localized stresses in thin films. We discuss the prospects of this technique for precision dynamic tuning of nanoscale opto-electro-mechanical devices and the development of field-programmable non-volatile set-and-forget architectures. We also outline some of the key challenges that need to be addressed with a view toward incorporating electrochemical stress tuning methods for post-processing foundry devices.

## Graphical abstract

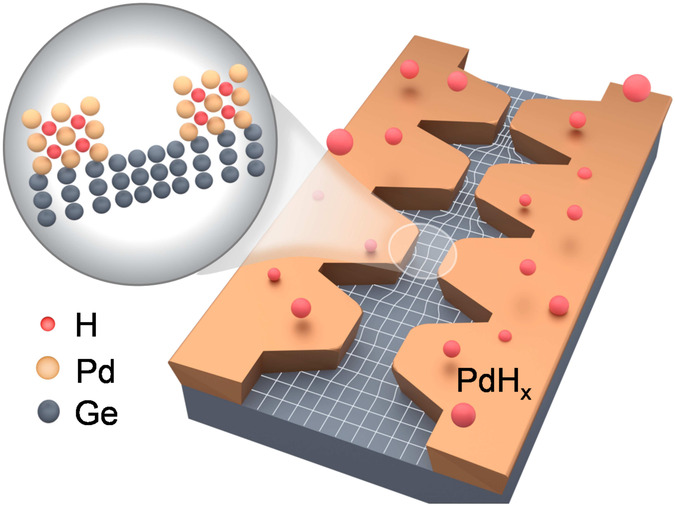

Exerting exquisite control over propagating fields in nanoscale (sub-micrometer) geometries using stress (and the associated strain) has been a unifying theme across diverse research fields, ranging from microelectronics^1^ and integrated photonics^2^ to microwave acoustics.^3^ The two hallmark examples of its impact on modern life are the use of stress to improve threshold current density in semiconductor quantum well lasers that power modern fiber-optic communication^4^ and improving channel carrier mobility in nanoscale transistors.^5^ These examples also illustrate how stress provides a universal tuning knob that can simultaneously manipulate multiple fields at the nanoscale, with a view toward controlling interactions in multi-resonant geometries, as is the case in devices such as a piezoelectric microwave to optical quantum transducers^6^ that rely on resonant acousto-optic interactions in nanoscale cavities.

A common feature among all existing approaches to applying stress in integrated platforms, whether actuated using thermal, piezoelectric,^7^ electrostatic,^8^ or magnetostatic^9^ effects, is that they are well-adapted to applying uniform stresses or fixed stress gradients. However, there are scenarios in which one ideally wants to control the 2D stress pattern at the nanoscale. These include the use of nansocale strain to create giant pseudo-magnetic fields in graphene^10^ and overcoming nanofabrication-induced variations^11–13^ in coupled cavity arrays for exploring many-body physic analogues of condensed matter phenomena.^14^ The ultimate goal is to be able to precisely control both the magnitude and sign (whether compressive or tensile) of the local stress at a given location with the flexibility to extend this spatial control arbitrarily over the 2D plane. The limitations of existing approaches mainly stem from either residual crosstalk (thermal effects) or the footprint needed for the actuator (almost all of the nanoelectromechanical system approaches). A second related theme is the need for methods that can implement set-and-forget tuning approaches that alleviate the need for an active tuning field. This becomes especially important when one is tuning large-scale resonant systems, a recurring theme in experimental implementations of quantum computing at scale in power- or footprint-constrained cryogenic environments.

Here, we explore the use of localized electrochemical absorption of hydrogen (H) in structured palladium (Pd) electrodes as a way to achieve localized control of stress with flexibility over engineering the stress pattern over the 2D plane. This enables the prospect of engineering field-programmable set-and-forget architectures with a view toward large-scale control of resonant nano-opto-electro-mechanical (NOEM) systems without requiring active tuning of each element. While the electrochemical absorption of hydrogen in palladium films is well-understood,^15^ this work builds on two key observations: (a) the hydrogen-loading fraction in the electrode can in principle be controlled with high precision, and as that determines the hydrostatic volume expansion directly, one has a way to program local strains precisely; (b) the spatial profile can be controlled by shaping the electrode through standard lithographic techniques. From a footprint perspective, the shaped Pd electrode functions as a nanoscale stress actuator and therefore provides a natural route to efficiently interface with NOEM devices.

We use a thin film of germanium (Ge) (1.6 μm) epitaxially grown^16^ on a silicon (Si) substrate as the test platform for proof-of-principle experiments for validating our ideas. The choice of Ge is mainly motivated by the prospect of using large tensile strains (≈2%) to engineer a transition from being an indirect bandgap semiconductor to one with a direct bandgap in a silicon-compatible material platform,^16,17^ which has potential benefits from an optoelectronics perspective, but the idea can be applied to any material platform of interest. In a way, one of the main advantages of this stress tuning method is the generality with which it can be applied to any potential NOEM platform being studied. PdH was chosen mainly for its simplicity, effectiveness of the induced volume expansion, and wide compatibility with device platforms, but the technique is broadly applicable to other electrochemical systems.

Figure 1 shows a schematic illustration of the technique. Structured Pd electrodes (*t*_Pd_ = 200 nm) are lithographically patterned by using standard electron beam lithography and lift-off procedures. A representative set of fabricated devices is shown in Figure 2a. We use a 2.5 nm chromium (Cr) layer underneath to maintain adhesion of the Pd film to the underlying substrate. Hydrogen is then electrochemically absorbed into the electrodes, resulting in hydrostatic volume expansion that can be controlled by the [H/Pd] ratio. Structuring the electrode shape allows us to exert local control over the stress, as shown in the inset of Figure 1, which shows how the stress at the center of the electrode gap scales with a reducing gap width. These two parameters, [H/Pd] ratio and geometric shape, provide two (independent) degrees of freedom for engineering the stress over the 2D plane.

**Figure 1:**
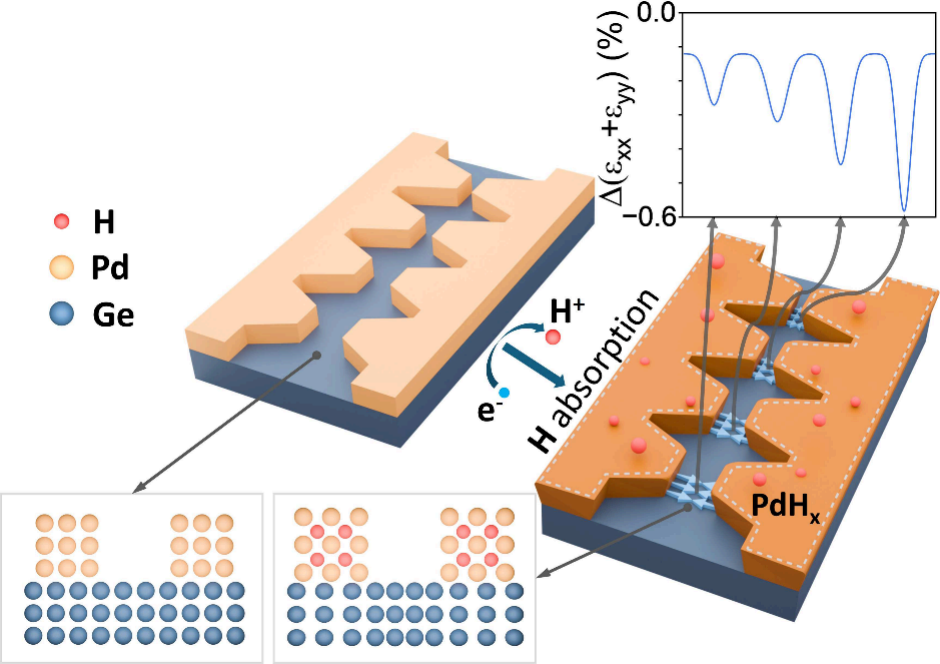
Schematic illustration of electrochemical absorption-induced localized stress generation. Pd electrodes are lithographically patterned on a Ge-on-Si substrate. Hydrogen is electrochemically absorbed into the Pd electrodes, resulting in a hydrostatic volume expansion that scales with the [H/Pd] ratio. This expansion exerts a localized stress on the underlying Ge film, and its magnitude can be controlled by choosing the electrode shape. The inset (top right) illustrates how the compressive stress registered at the center between the electrodes scales with a reducing electrode gap.

**Figure 2:**
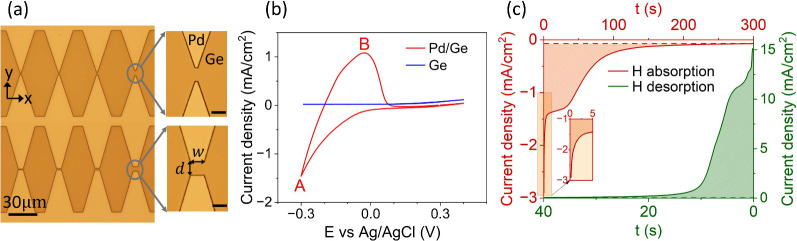
(a) Optical microscope images of structured 200 nm thick Pd electrodes on the Ge-on-Si substrate (scale bar for zoomed-in insets is 5 μm). (b) CV curves for Ge on Si with and without a Pd electrode in 0.1 M H_2_SO_4_ (scan rate of 50 mV s^–1^). (c) CA curves for H loading at −0.26 V (red, left *y* axis and top *x* axis) and desorption at 0.24 V (green, right *y* axis and bottom *x* axis) with dashed red and green lines indicating the background currents.

Figure 2b shows the cyclic voltammetry (CV) curves of Ge, with and without the Pd electrode, obtained in 0.1 M H_2_SO_4_ at a scan rate of 50 mVs^–1^, using Ag/AgCl as the reference electrode and a Pt wire as the counter electrode. No peaks were observed for the bare Ge sample, indicating that no redox reaction occurred on the Ge surface. Peak A on the curve of the sample with the Pd electrode corresponds to hydrogen absorption (H^+^ + e^–^ → H_abs_) and the hydrogen evolution reaction (2H^+^ + 2e^–^ → H_2_), while peak B corresponds to desorption (H_abs_ → H^+^ + e^–^).^18^ Before the stress induced due to H loading was quantified, the sample was cycled until repeatable CV behavior was obtained; see section 1 of the Supporting Information for additional details.

Hydrogen absorption in the palladium film was carried out using the potentiostatic technique with chronoamperometry (CA) curves shown in Figure 2c, where a constant reduction potential (−0.26 V vs Ag/AgCl) was applied for hydrogen loading, followed by an oxidation potential (0.24 V vs Ag/AgCl) for desorption. The volume expansion scales with the atomic ratio of [H/Pd]. The number of absorbed hydrogen atoms was derived from the reduction/oxidation charge by integrating the chronoamperometry absorption/desorption curve with the background current removed, and the number of palladium atoms was calculated from the geometric volume (electrode area × film thickness) and density of the Pd film. We measure the charge densities *Q*_abs_ = −139 mC cm^–2^ and *Q*_des_ = 138 mC cm^–2^ for a [H/Pd] loading ratio of 0.6, calculated from a sample surface area of 0.8 cm^2^.

We use a micro-Raman spectrometer (Renishaw InVia) to quantify *in situ* the local strain induced by the volume expansion of the Pd electrode on the Ge film. The laser excitation wavelength in the system is 488 nm, and it is equipped with a three-axis microscope stage enabling a mapping step size of 100 nm. The laser beam is focused on the sample through an objective lens with a numerical aperture (NA) of 0.6 and a 50× magnification, resulting in a spot size of ≈700 nm on the sample surface, while being focused through an electrolyte. Given that the absorption length in Ge at the laser wavelength is ≈20 nm, the Raman (strain) signals reported here should be interpreted as the effective strain being induced in the top 10 nm of the Ge film.

From the measured Raman peak shift ν, the strain induced in Ge relative to the unstrained Ge film can be derived from the Raman frequency shift $\Delta \nu =\nu -\nu_{0}=b\frac{\left(\varepsilon_{xx}+\varepsilon_{yy}\right)}{2}$ , with *b* = [*q* – *p*(*C*_12_/*C*_11_)]/ν_0_. Here, ν_0_ is the frequency shift for bulk Ge (300 cm^–1^);^19^ *p* and *q* are the phonon deformation potentials; and *C*_11_ and *C*_12_ are the elastic constants of Ge. A value of *b* = (−415 ± 40) cm^–1^ was employed in this study.^20^ To calibrate our measurements, we find a 0.2% biaxial tensile strain using this approach on the unpatterned Ge film, which is consistent with the reported film strain due to thermal expansion mismatch between Ge and Si.^16,21^ Since the strain within the electrode gap induced by the Pd expansion is anisotropic, being predominantly compressive in the *y* direction (cf. Figure 2a), with a minor tensile component in the *x* direction (shown in Figure S7c and d of the Supporting Information), the strain is herein defined as the sum of the in-plane strain components (ε_*xx*_ + ε_*yy*_). The strain change Δ­(ε_*xx*_ + ε_*yy*_) in the following discussions refers to the net in-plane strain change relative to the Ge film with Pd electrodes before any electrochemical experiments are performed. We also assume that the *x* and *y* axes in Figure 2a correspond to the [100] crystal axis of Ge.

Figure 3a shows a representative Raman shift measurement to map the strain at the center of the electrode gap, as shown in Figure 2a, with parameters *w* = 5 μm and gap *d* = 500 nm. The black curve shows the measured shift of the device pre hydrogen loading; the blue curve is the shift with [H/Pd] = 0.6; and the red curve is taken after H is desorbed from the device. The hydrostatic expansion-induced Raman shift is clearly visible. As expected and confirmed by finite element method (FEM) simulations in Figure S7 in section 2 of the Supporting Information, we observe a compressive strain in the region between the electrodes due to hydrostatic expansion in the electrodes themselves. To reiterate, the Ge film underneath the electrodes, which we cannot access in this measurement, is tensile-strained (Figure S8 of the Supporting Information), but the interelectrode gap (cf. Figure 2a) is compressively strained.

**Figure 3:**
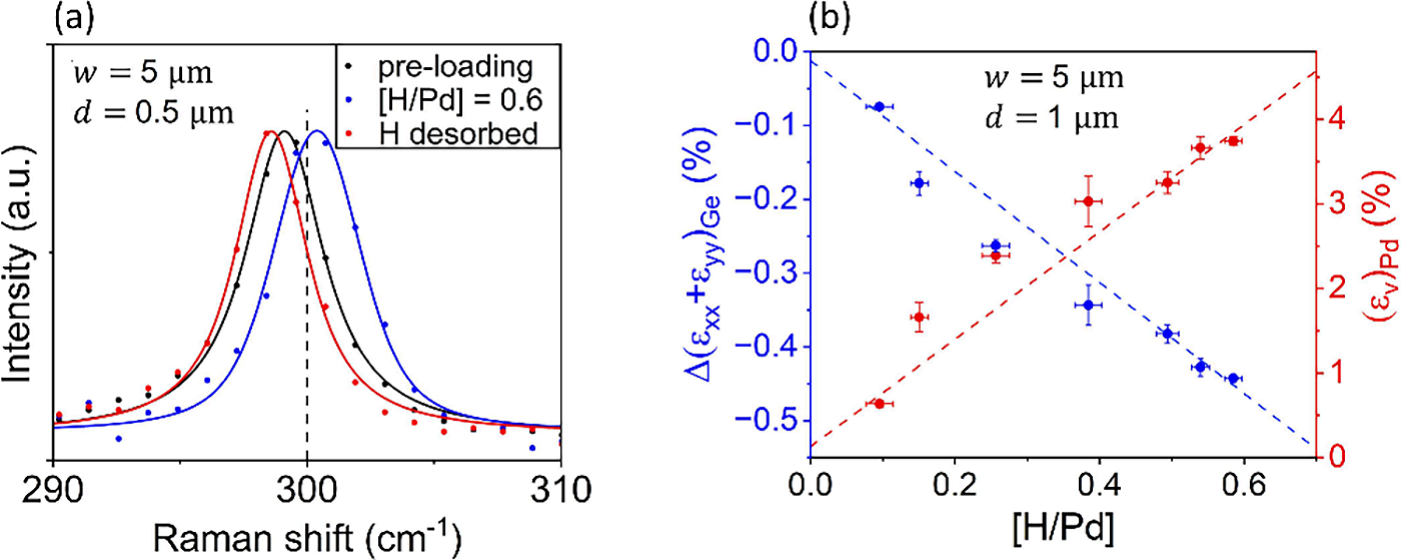
(a) Raman measurement of stress induced in a Ge-on-Si film due to the hydrostatic expansion of a structured Pd film due to H absorption. The black curve shows the measured Raman shift of the bare film with Pd electrodes preloading; the blue curve shows the shift with a [H/Pd] loading ratio of 0.6; and the red curve shows the shift once H is desorbed. All measurements are made at the same location at the center of the electrode with parameters *w* = 5 μm and *d* = 500 nm (cf. Figure 2a). The compressive stress induced by the expansion is clearly seen. The Raman shift of unstrained Ge is shown by the dashed line. (b) Left *y* axis shows the induced in-plane strain change as a function of the H-loading fraction. The strain is extracted from the Raman shift as shown in panel a for the Ge film at the center of the electrode gap, as indicated in Figure 2a for a device with parameters *w* = 5 μm and gap *d* = 1 μm. The compressive strain increases approximately linearly (blue dashed line) with the loading fraction as expected. Using FEM modeling, one can use the measured strain to infer the volumetric expansion of the Pd electrodes, which is shown with the right *y* axis.

Figure 3a also shows that the H loading and unloading process irreversibly transforms the Ge surface, indicated by the red curve (post H desorption) not lining up with the black one (preloading H). We do not currently understand how the Ge surface is exactly modified after the repeated stress and destress cycles, but as we show in section 3 of the Supporting Information, the loading and unloading cycles do cause defect formation analogous to what has been observed in thermal cycling of Ge films between room temperature and 850 °C.^22^ We also note that the considerable lattice strain induced in the Pd film due to absorption of H in Pd near the α → β phase transition of PdH_*x*_ has been shown to result in defects and limited reversibility of Pd film-based actuators, which could potentially be playing a role here as well.^23,24^

Since the stress is controlled by the volumetric expansion of the Pd electrodes, which is determined by the [H/Pd] ratio, one can in principle program the stress precisely by controlling the [H/Pd] ratio. Figure 3b shows the measured in-plane strain change Δ­(ε_*xx*_ + ε_*yy*_), which can be inferred from the Raman shift as a function of the [H/Pd] loading fraction. The different data points in Figure 3b were taken over time during potentiostatic hydrogen absorption. Each data point is an averaged value of five repeated Raman measurements taken at the center of the electrode gap with parameters of *w* = 5 μm and *d* = 1 μm. The uncertainty in the [H/Pd] ratio arises from both the Raman signal acquisition time (≈10 s) and uncertainty in the overall size of the electrode over which the H loading occurs.

To get the volumetric strain ε_v,Pd_, defined as the relative volume change (Δ*V*/*V*_0_) in the Pd electrode (right *y* axis in Figure 3b), we iteratively adjust the strain in the Pd layer in simulation (see section 2 of the Supporting Information), until we match the volume-averaged (laser spot size × absorption depth) strain change in the Ge film at the center of the electrode. In the simulation, a background strain change (ε_bkgd_) of −0.1% for [H/Pd] = 0.6 was applied based on the measurement at a location of (10 μm) far from the interelectrode gap to get better agreement between simulations and experiment by considering the difference between model dimension and the actual sample size (see section 2 of the Supporting Information for more details). For instance, for electrode parameters *w* = 5 μm and *d* = 1 μm, with an ε_bkgd_ of −0.1% for a [H/Pd] loading ratio of 0.6, we find an ε_v,Pd_ of 3.7%. This value is about 3× lower than the 11% lattice volume strain reported for both bulk Pd^25^ and a clamped Pd film^26^ for a similar [H/Pd] loading. However, it is important to keep in mind that, for our structured films, the in-plane macroscopic expansion is more severely limited by the substrate constraint, resulting in an increased compressive stress and larger out-of-plane expansion in the Pd film. Using our simulations, we estimate the in-plane compressive stress in the Pd electrode, which we cannot access in the Raman experiment directly, and we find it to be ≈ –1.6 GPa at [H/Pd] = 0.6 (Figure S5 of the Supporting Information), which is in reasonable agreement with the reported value for a clamped film at a similar [H/Pd] loading.^26^ Since we measure the residual background strain only at [H/Pd] = 0.6, the background corrections for the other [H/Pd] loading ratios in Figure 3b used to generate ε_v,Pd_ are linearly interpolated; therefore, an ε_bkgd_ = −0.05% is used for [H/Pd] = 0.3.

Figure 4 shows the effect of the electrode shape on controlling the induced strain for a fixed [H/Pd] loading (constant ε_v,Pd_). It plots the 1D line-cut *x* profiles of the measured and simulated strain at the center of the interelectrode gap, shown by dashed lines in Figure 4a, for different electrode shapes (varying gap *d* and width *w*) at a fixed [H/Pd] of 0.6. For a fixed gap *d*, the strain can be increased by increasing width *w*, and for a fixed gap *w*, the strain can be increased by reducing *d*. One can use this procedure to generate look-up tables for induced strain vs geometry for a given [H/Pd] ratio and then use this as a programmable knob to engineer the desired strain at given location in the 2D plane.

**Figure 4:**
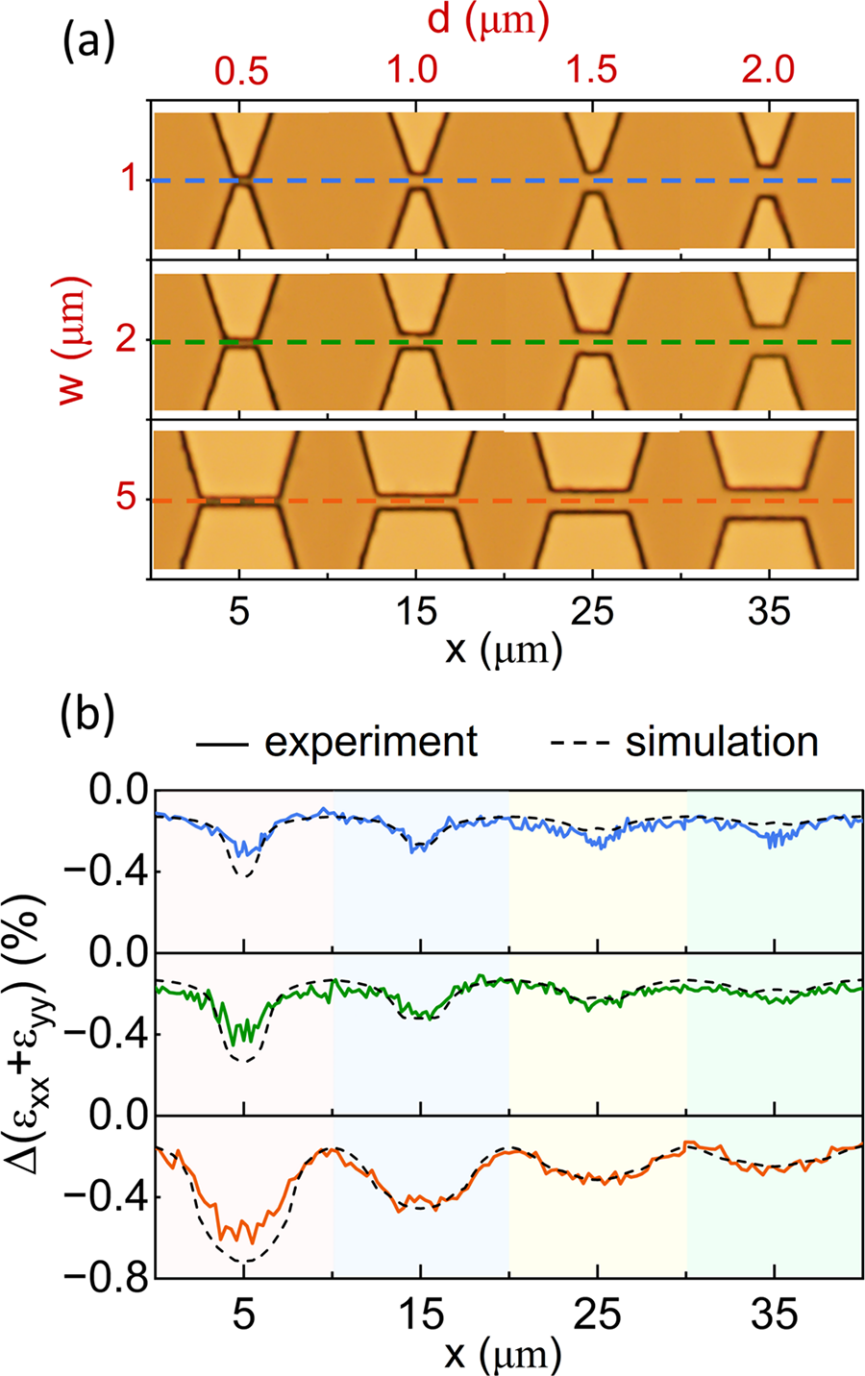
Controlling the 2D strain profile through varying the electrode shape for a fixed [H/Pd] loading ratio of 0.6. (a) Optical micrograph of fabricated devices with fixed electrode widths *w* of 1 μm (top), 2 μm (middle), and 5 μm (bottom row) and varying electrode gaps *d* ranging from 0.5 to 2 μm. (b) 1D strain maps (generated from the corresponding Raman data) of the devices from panel a taken across the center of the interelectrode gap. The effect of the electrode geometry on the induced strain can be clearly seen and can be reproduced well from FEM simulations (dashed black curves). The discrepancy for the *d* = 0.5 μm cases is discussed in the main text.

When the electrode gap *d* becomes smaller than the spot size in our experiment (700 nm), we find that the measured strain is significantly lower than what the simulation predicts. This is clear in all of the results for *d* = 0.5 μm in Figure 4b. We believe that the discrepancy arises from the change in the effective spot size on the Ge surface due to both the narrow gap (metal occlusion) and the expansion of the Pd electrode. We tried to account for this in our simulation by reducing the effective spot size, which was used to compute the volume-averaged strain. The dashed black simulation curve for *d* = 0.5 μm in Figure 4b was calculated using an effective spot size with a width of 200 nm along the *y* axis and 700 nm along the *x* axis (cf. Figure 2a). Even with this reduction, there is a significant discrepancy where the measured strain is below what the simulation predicts. If we use a spot size of 500 nm along the *y* axis corresponding to the actual electrode gap and the predicted laser spot size (700 nm) along *x*, the simulated compressive strain is ≈ –0.95%, significantly above what we measure experimentally (≈ –0.62%).

The electrode geometry in Figure 4a exerts primarily a compressive stress along the *y* direction (cf. Figure 2a) in the gap region. By adding a second pair of electrodes along an orthogonal direction (Figure 5), one can exert a biaxial compressive strain in the gap region and effectively double the induced in-plane strain. Assuming that we limit the overall applied strain to operate within the linear regime, we can choose the in-plane electrode geometry to locally modify the stress and significantly enhance it by geometric stress concentration. An implementation of this idea is shown in Figure 5, which shows the progressive increase in measured in-plane strain due to varying the electrode geometry. The left side of Figure 5a, c, and e shows the strain pre H loading, and the right side of Figure 5b, d, and f shows the strain for a loading [H/Pd] = 0.6 for different geometries. Moving from a point electrode (*w* = 0) (b) to our standard reference electrode (*w* = 1 μm) (d), the compressive strain in the gap increases, reproducing the results from Figure 4. Adding a second electrode with (*w* = 1 μm) oriented orthogonally to the first (f) exerts a biaxial compressive strain due to expansion, and the resulting in-plane strain is significantly enhanced (≈2×) to −0.8%. We would like to note that, due to electrode symmetry, the strain is truly biaxial only at the exact center of the gap.

**Figure 5:**
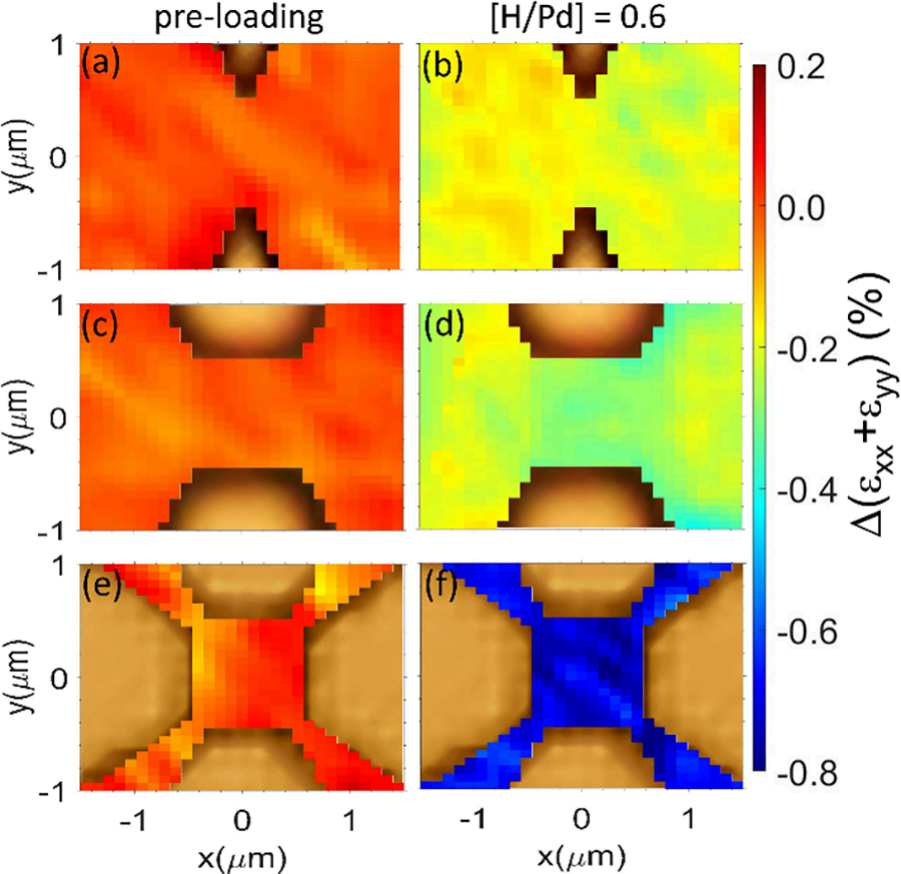
Strain concentration and enhancement via geometry. Since the Raman shift is sensitive to the total in-plane strain (ε_*xx*_ + ε_*yy*_), one can shape the electrodes in order to exert a biaxial compressive strain, which should double the overall measured strain. (a) Electrode shaped to a point tip with *w* = 0 and *d* = 1 μm, (b) standard electrode as discussed in Figure 3 with *w* = 1 μm and *d* = 1 μm, and (c) squared gap electrode geometry with electrodes along both the *x* and *y* axes with *w* = 1 μm and *d* = 1 μm. Doing a 2D strain scan (via the Raman shift) pre H loading (left panel) and with [H/Pd] = 0.6 (right panel) clearly shows the strain enhancement (≈2×) effect in the squared gap geometry in panel c compared to the reference case in panel b.

There are a few challenges that need to be addressed if this electrochemical stress tuning technique is to be widely deployed for tuning NOEM devices. While we have clearly shown that the combination of geometry with the [H/Pd] loading ratio can be used as a platform for tunable strain generation, we have not demonstrated precision control. The exquisite control over the [H/Pd] loading ratio, which is needed for precision stress engineering, is possible in principle but is limited in our experiment by the choice of 0.1 M H_2_SO_4_ as the electrolyte and unavoidable presence of oxygen traces in the solution. The [H/Pd] loading ratio is extremely sensitive to potential (Figure S4 of the Supporting Information) due to the α → β phase transition of PdH_*x*_. Moving to a different electrolyte, like an ionic liquid-based electrolyte,^27^ will make this programming easier. In addition, applying an ultrathin silicon oxide overlayer can help mitigate the oxygen reduction reaction (ORR),^28^ thereby improving the precision of the [H/Pd] ratio and thus the precision of stress control by reducing the influence of parasitic reactions.

A second limitation, especially with a view toward field-programming stress in NOEM devices, is that our experiments are performed *in situ* (section 1 of the Supporting Information). We can only maintain H inside Pd in an active electrochemical cell. As soon as the electrolytic reaction stops and the electrolyte is removed, H desorbs from the Pd film and the applied stress is removed as Pd relaxes back. For this technique to successfully interface with NOEM devices, H needs to be trapped inside Pd once a desired loading has been reached. This is critical for set-and-forget architectures, where a desired strain can be programmed and maintained in a non-volatile fashion. While achieving non-volatile stress retention remains an active challenge in the current system, the use of sulfur to encapsulate the Pd film through surface poisoning has shown potential in this regard and is a key component of future studies.^29,30^

The other open questions that need to be addressed with NOEM devices in mind are nanoscale electrode actuation and the maximum stresses that can be applied using this method. Nanoscale electrode actuation can in principle be achieved by using the (doped) semiconductor as the electrode, although the effectiveness of this technique needs to be demonstrated in practice. With regard to the strain limits, the two key parameters that one can control experimentally, ignoring any strain enhancements via geometry, are the [H/Pd] loading ratio and the thickness of the Pd film. While, in this work, we saturate the H loading at 0.6, Pd in principle can approach loading ratios of >0.8.^31^ Increasing the Pd film thickness for a constant [H/Pd] ratio also increases the induced strain. Figure S6 of the Supporting Information shows the increase in the in-plane strain for the standard electrode geometry, shown in Figure 2a, for device parameters *w* = 5 μm and *d* = 1 μm. The net strain approaches −0.7% for the single electrode case with a Pd thickness of 1 μm. In practice, we expect to keep the Pd film thickness below 500 nm in order to be compatible with NOEM devices.

## Supplementary Material



## Notes

*In situ* Raman measurement (setup and data acquisition) (section 1), strain modeling using FEM simulations (section 2), and formation of defects (section 3) (PDF)
