# Supplementary material for: Exploring Electrochemical Methods for Precision Stress Control in Nanoscale Devices
Source: Nano Lett. 2025 Aug 13;25(34):12899–904. doi: 10.1021/acs.nanolett.5c02870 (PMC12395479; doi:10.1021/acs.nanolett.5c02870)
Supplement: Supplementary file 1 [file nl5c02870_si_001.pdf]

Supporting Information

Exploring electrochemical methods for precision stress control in nanoscale devices

Di Chen,*,† Natasa Vasiljevic,† Andrei Sarua,‡ Martin Kuball,‡ and Krishna C. Balram*,¶

†*School of Physics, H.H. Wills Physics Laboratory, University of Bristol, Bristol BS8 1TL, United Kingdom*

‡*Centre for Device Reliability and Thermography, School of Physics, H.H. Wills Physics Laboratory, University of Bristol, Bristol BS8 1TL, United Kingdom*

¶*Quantum Engineering Technology Labs and School of Electrical, Electronic and Mechanical Engineering, University of Bristol, Bristol BS8 1UB, United Kingdom*

E-mail: di.chen@bristol.ac.uk; krishna.coimbatorebalram@bristol.ac.uk

1. In situ Raman measurement: setup and data acquisition

The in situ Raman measurements were conducted in 0.1 M H_2SO_4 using the setup shown in Figure S1 with Ag/AgCl as the reference electrode and a Pt wire as the counter electrode. The electrical contact on the sample was made using copper wire, sealed from the electrolyte with nail polish. The sealed area was minimized to maximize the accessible Pd film area, and the associated error was included in the geometric uncertainty for the [H/Pd] loading ratio

calculation. During the Raman measurements, the laser power was adjusted to avoid heating on the sample and minimizing the effect on the measured current. A laser power of 45 mW was applied: 1% power with an acquisition time of 8 s for measurements in air, and 10% with an acquisition time of 2 s in the electrolyte. Figure S2 shows the Raman shifts (three accumulations of each curve) of the bare Ge substrate, the Ge substrate with patterned Pd electrodes, the sample in electrolyte, and after CV cycling. The last three measurements were taken from the same sample at the same position, confirming the stability of the Ge layer throughout these steps. Although the fabrication-induced strain($\pm$ 0.1%) in Ge film was observed in some samples, it was mostly localized at the edges of Pd electrodes. To minimize these edge effects, most of the measurements reported in this work with the exception of the 2D strain maps, were taken at or along the centre of the electrode gaps.

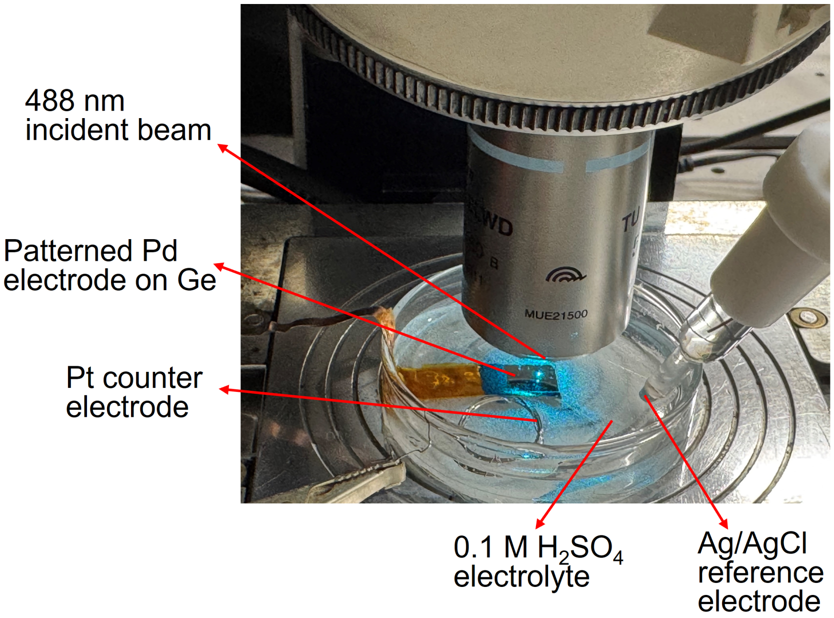

Figure S1: In situ Raman measurement setup: the sample is mounted in a home built electrolytic cell which can be mounted on the sample stage of the Raman spectrometer and fits within the working distance of the objective. The sample, electrodes and electrolyte are indicated.

The Raman data with hydrogen involved were collected after the first loading/unloading cycle as a non-linear strain-[H/Pd] relationship was observed during the initial loading, which we attribute to plastic deformation in the Pd electrode,[1] and the formation of defects in the

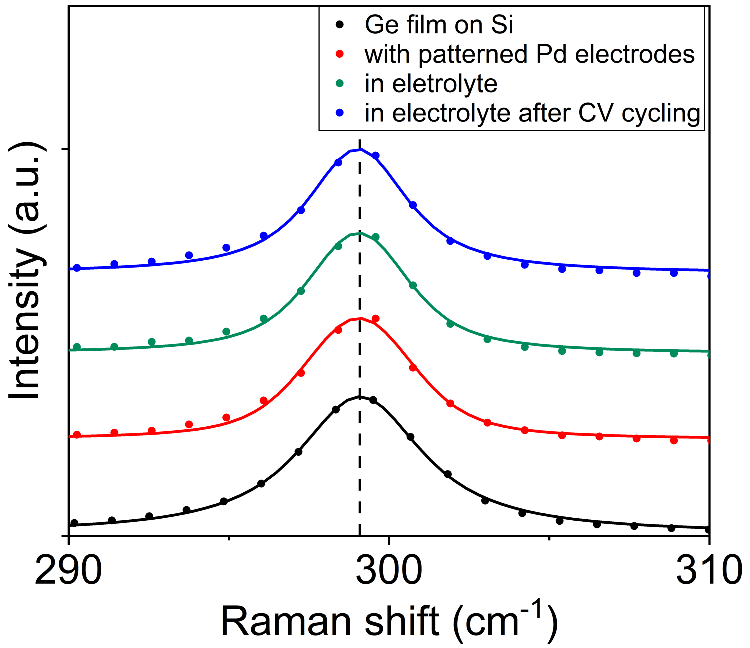

Figure S2: Raman measurements of the bare Ge-on-Si substrate, the Ge-on-Si substrate with patterned Pd electrodes, the sample in electrolyte, and after CV cycling. The last three measurements were taken on the exact same location.

Ge layer during the first loading. The strain response was consistent from the second loading onward, corresponding to that shown in Figure 3(b) in the main text, and no newly formed defects were observed in Ge layer as long as the [H/Pd] ratio was kept below 0.6. In our experiments, strain measurements were performed for 10 cycles, during which both the strain response and the amount of absorbed hydrogen in Pd film remained reversible. For stress tuning, the system in general does not need to withstand numerous cycles as in hydrogen separation or sensing use cases. Rather, the objective is to tune the [H/Pd] ratio to induce the desired stress, so the reversibility over about 10 cycles is sufficient.

On the other hand, when we tried to increase the [H/Pd] ratio to ≈ 0.7 in 0.5 M H_2SO_4, we observed a drop in the compressive strain during the fourth loading, accompanying the formation of new defects in the Ge layer. We discuss this further in Section 3 below. Figure S3 shows the measured Raman shifts for intermediate [H/Pd] loading ratios. This data was used to generate the strain plot shown in Figure 3(b) in the main text. Figure S4 shows the dependence of [H/Pd] ratio on the absorption potential. Each absorption potential was

applied for a sufficiently long time, at least 10min, until a stable background current was obtained. The saturated [H/Pd] ratios were quantified using the oxidation charges from desorption CA curves.

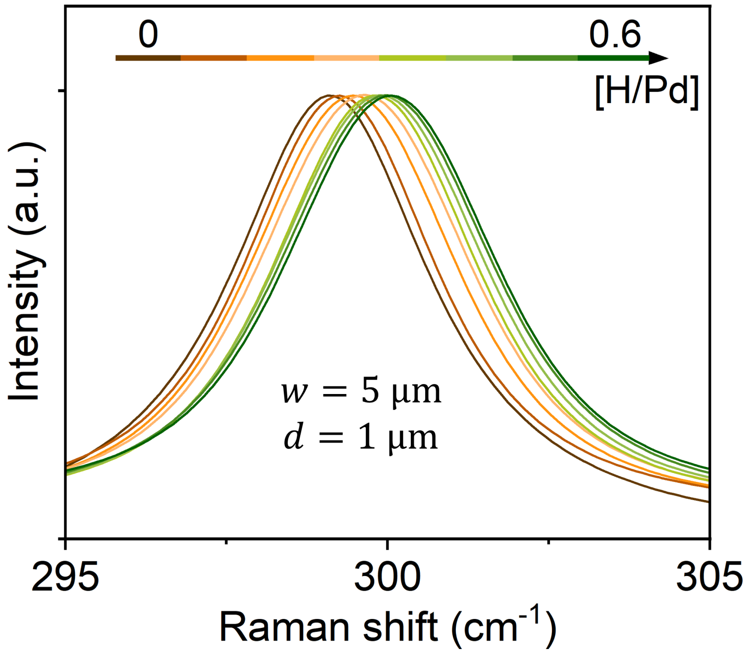

Figure S3: Measured Raman shifts for the standard electrode geometry $w = 5\,\mu\text{m}$ and $d = 1\,\mu\text{m}$ for intermediate [H/Pd] loading ratios. This data underlies the estimated strain values reported in Figure 3(b) in the main text.

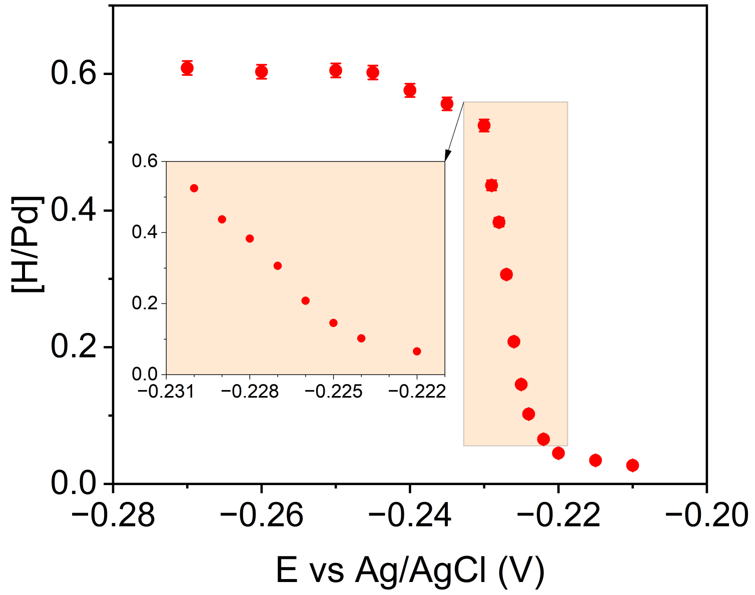

Figure S4: The dependence of [H/Pd] ratio, quantified through the oxidation charge from desorption CA curve, on the absorption potential. Each absorption potential was applied for at least 10 min until a stable background current obtained. Precision stress tuning using this technique requires precision control over the [H/Pd] loading ratio, which is limited in our experiment by the steepness of the loading curve with voltage, see inset.

2. Strain modelling using FEM simulations

The FEM modelling, performed using COMSOL Multiphysics, consisted of two steps: first, we iteratively estimate the volumetric strain ($\varepsilon_{v,Pd}$) of the Pd electrode by reproducing the strain measured from the Ge film at the centre of electrode gap ($w = 5\,\mu$m, $d = 1\,\mu$m); then, we fix this extracted $\varepsilon_{v,Pd}$ and use it as an input (pre-stressed) to model the strain profiles that can be generated in other devices by varying electrode shapes. To compare the simulated strains with the strain extracted from the Raman measurements, an absorption volume of the laser (spot size $\times$ penetration depth) was estimated to calculate the average strain in the simulation. The 488 nm laser beam is focused on the sample through an objective lens with a numerical aperture (NA) of 0.6 and a 50$\times$ magnification, resulting in a spot size of $\approx$ 700 nm on the sample surface. This value is calculated from spot diameter $d = \frac{2\lambda}{\pi \cdot NA}$ with an assumed magnification factor of 1.4 for the measurements in electrolyte. The optical probing depth in Ge, relevant for the Raman measurements, is $\approx$ 10 nm.

The 1.6 µm thick Ge layer and the Si substrate were defined as anisotropic elastic materials, while the 200 nm Pd electrode and 2.5 nm Cr adhesion layer were defined as isotropic. The dimensions of model were 20 µm in width (x-axis) and 60 µm in length (y-axis), referred to the coordinate system in Figure 2(a), with a fixed constraint applied to the bottom surface of the Si substrate and all other surfaces free to deform. The coordinate origin was defined as the centre of electrode gap on Ge surface and we assume that the coordinate axes are oriented along [100]. A background strain change of -0.1% was applied to the simulations for [H/Pd] = 0.6 to get better agreement between simulations and experiment as we measure residual strain at a location of (10 µm) far from the inter-electrode gap. The strain there in theory (and predicted by the FEM simulation) is nearly zero and this background strain comes from the effect of the unpatterned Pd electrode which surrounds the structured Pd region like a frame and is needed for maintaining electrical connectivity to drive the electroabsorption process. Because of computational restrictions, we do not model this frame and instead add a background strain to our simulations. The background strain for other [H/Pd] loading ratios reported in Figure 3(b) was linearly extrapolated from the -0.1% at [H/Pd] = 0.6. Figure S5 shows the derived biaxial stress in the Pd electrode (w = 5 µm, d = 1 µm).

For the simulations to study the dependence of strain on Pd thickness (t_{Pd}) shown in Figure S6, and strain profiles of device shown in Figure S7, the background strain was excluded in line with our long-term objective of using nanoscale Pd electrode actuators strain engineering. Figure S7 shows the simulated strain profiles of a device with w = 5 µm, d = 1 µm with all layers. Figure S7(b), (c), (d) show the zoomed-in strain profiles of $\Delta(\varepsilon_{xx} + \varepsilon_{yy})$, $\Delta\varepsilon_{xx}$ and $\Delta\varepsilon_{yy}$ of Ge layer sliced along $x = 0$ and $y = 0$, indicated by dashed lines in Figure S7(a). At the centre point of the electrode gap, $\Delta(\varepsilon_{xx}+\varepsilon_{yy})$ = -0.30%, $\Delta\varepsilon_{xx}$ = 0.06% and $\Delta\varepsilon_{yy}$ = -0.36% from the simulation, indicating the tensile strain in $x-$ direction and compressive strain in $y-$ direction. The volume averaged strains calculated from laser absorption volume are $\Delta(\varepsilon_{xx} + \varepsilon_{yy})$ = -0.34%, $\Delta\varepsilon_{xx}$ = 0.06% and $\Delta\varepsilon_{yy}$ = -0.40%).

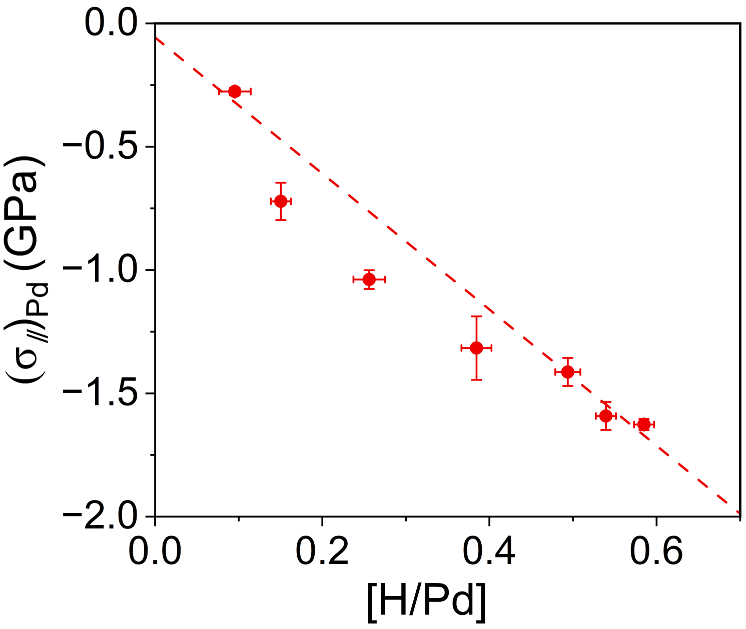

Figure S5: Predicted biaxial stress in Pd electrode (w = 5 µm, d = 1 µm) as a function of [H/Pd] ratio, iteratively derived from measured strain change $\Delta(\varepsilon_{xx}+\varepsilon_{yy})_{Ge}$ shown in Figure 3(b).

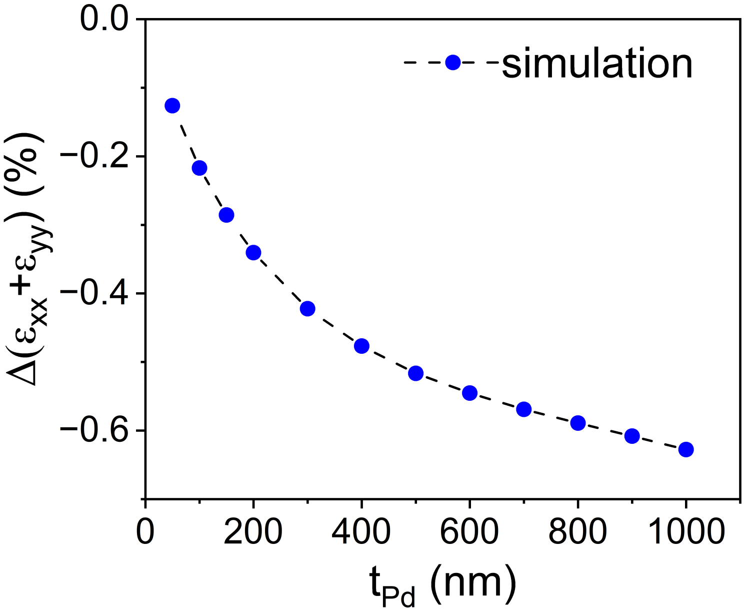

Figure S6: Dependence of the induced strain on the thickness of the Pd electrode for a fixed [H/Pd] = 0.6 estimated via FEM modelling. Simulations are carried out for the standard electrode geometry used in this work shown in Figure 2(a) with electrode parameters w = 5 µm and d = 1 µm.

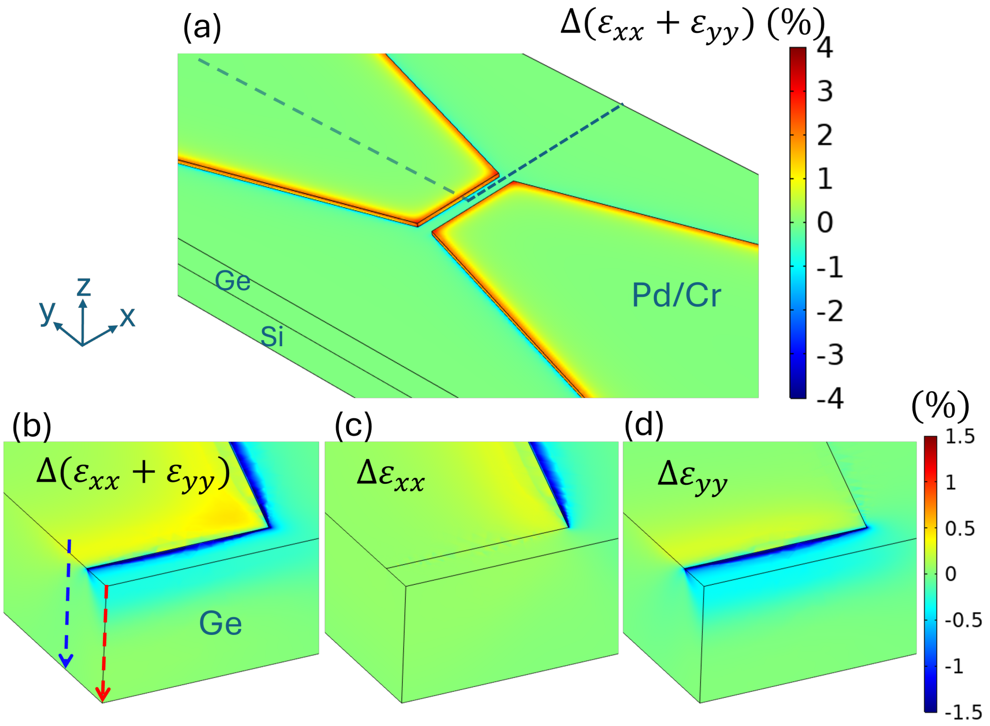

Figure S7: Simulated strain profiles with Pd electrode geometry of $w = 5\,\mu\text{m}$, $d = 1\,\mu\text{m}$ with the origin of the coordinate system defined as the centre of electrode gap on the Ge surface. (a) $\Delta(\varepsilon_{xx} + \varepsilon_{yy})$ distribution for all layers. (b) $\Delta(\varepsilon_{xx} + \varepsilon_{yy})$, (c) $\Delta\varepsilon_{xx}$ and (d) $\Delta\varepsilon_{yy}$ profiles in the Ge layer sliced along $x = 0$ and $y = 0$, indicated by the dashed lines in (a).

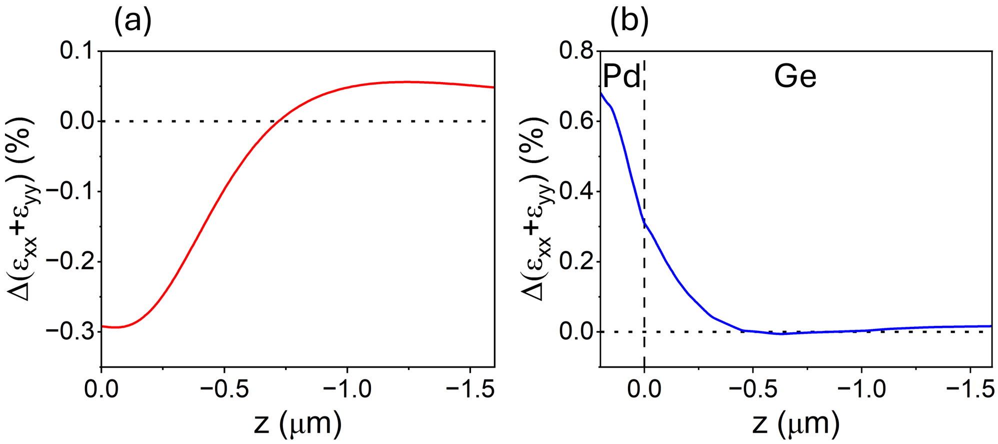

Figure S8: Evolution of strain with depth (z-profiles) shown by 1D strain cuts taken along the (a) red-dashed and (b) blue-dashed lines indicated in Figure S7(b). $z = 0$ is defined at the Ge surface. As noted in the main text, the in-plane strain under the electrodes is predominantly tensile, switching to compressive in the electrode-gap which is what we mainly measure in the Raman experiments.

Because of the penetration depth of the laser used in the Raman experiments, most of the strain values we report are effectively surface strains (depth $\approx 10\,\mathrm{nm}$). To get an estimate of the strain evolution with depth, we rely on our FEM simulation. Figure S8 shows the extracted $z-$profiles of strain (1D depth scan) along the dashed red and blue lines shown in Figure S7(b). $z = 0$ is defined at the Ge surface. Panel (a) corresponds to the red dashed line which is 1D strain depth scan taken at the centre of the electrode gap, and (b) the blue dashed line is measured $0.5\,\mu\mathrm{m}$ away from the electrode edge under the Pd electrode. As expected, the in-plane strain switches polarity from being tensile under the electrodes to being compressive in the gap. In the measurements, we can only access the strain in the Ge film in the gap.

3. Formation of defects

As noted in the main text, we observed defects forming in the Ge layer due to the repeated strain loading and unloading process. Figure S9 shows a representative microscope image of one of these defects formed in the Ge layer after the first (a) and sixth (c) loading/unloading cycles, giving a time-lapse image of defect evolution. Figure S9(b) and (d) present the corresponding thickness profiles measured using an optical profilometer, showing defect depth of approximately 1.6 µm, which matches the thickness of the Ge layer and shows that the defect there extends all the way to the Si substrate and the Ge is completely removed. This is further confirmed by the Raman measurement at the defect site from sample after the sixth loading/unloading cycle (Figure S10), which shows only the Si signal. As noted in the main text, similar defects are observed in temperature cycling Ge-on-Si substrates between room temperature and 850 °C,[2] which provides an indication of the stresses involved.

To better understand the enlargement of these strain induced fractures, we recorded the defect evolution during the loading/unloading cycles, as shown in Figure S11. The fracture was formed during the first loading with [H/Pd] = 0.3. From the resolution of our microscope images, it is hard to pinpoint the exact [H/Pd] ratio at which it starts forming. The enlargement of the defect occurred primarily during the unloading, continuing even after the electrochemical unloading had stopped. We believe these defect likely originate from point defects / threading dislocations in the Ge film which become energetically favored under applied stress. The Ge around the dislocations was preferentially removed due to the strain concentration at the defect. Since the stain is more concentrated at the electrode edges as shown in Figure S7(b), we find that these defects are more likely to form near the electrode edges, although we haven't statistically quantified this. We would like to note that the defects have a very specific crystalline orientation, as can be seen by their shapes, which also provides evidence for them becoming energetically favored under large applied stress, providing a mechanism for the film to relax. Moving to a membrane geometry provides additional routes for stress incorporation and potentially avoid these defects forming. In

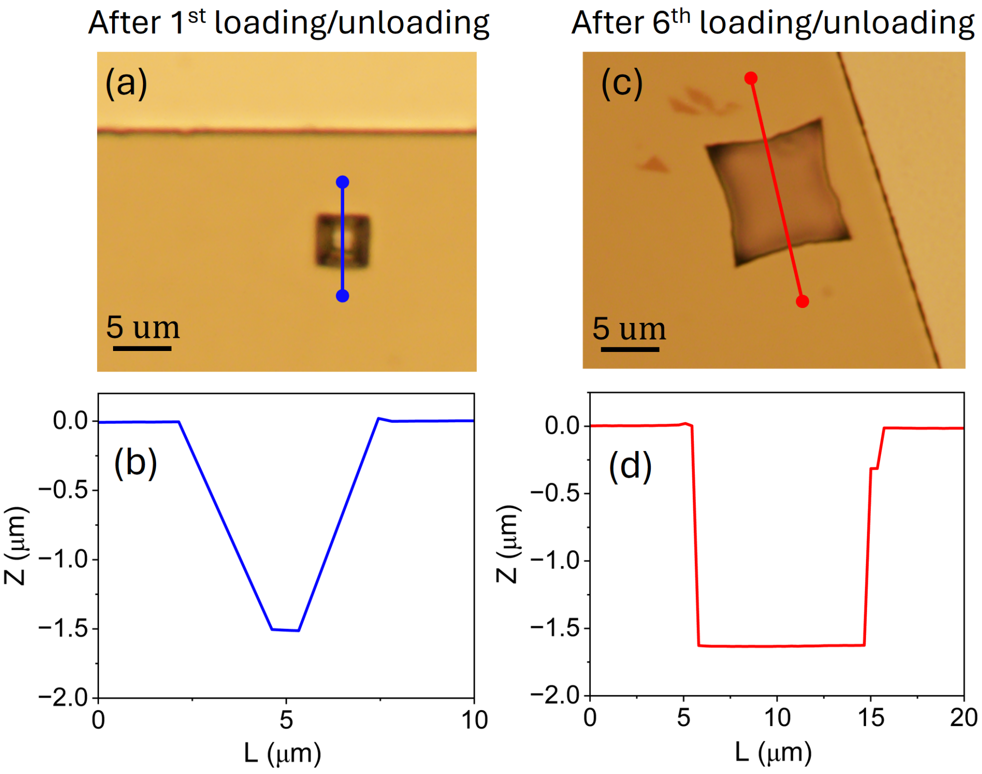

Figure S9: Optical microscope images of a representative defect formed in the Ge layer after the (a) first and (c) sixth loading/unloading cycle with thickness profiles along the blue (b) and red (d) line indicated underneath showing the Ge is effectively removed from that site.

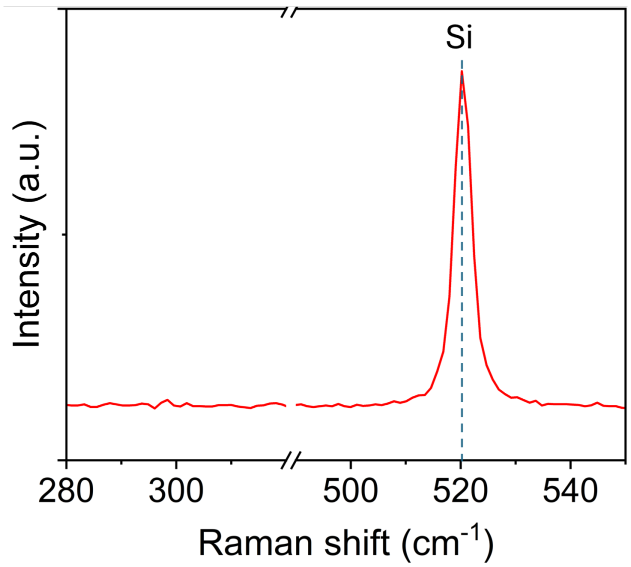

Figure S10: Measured Raman shift at the defect site shown in Figure S9 after the sixth loading/unloading cycle. We don't observe a Ge signal anymore further confirming that the Ge from that site is completely removed.

prior experiments with membranes[3,4] where similar strains were applied, we did not observe these defects although it is important to note that in those experiments the strain was fixed and not cycled unlike here.

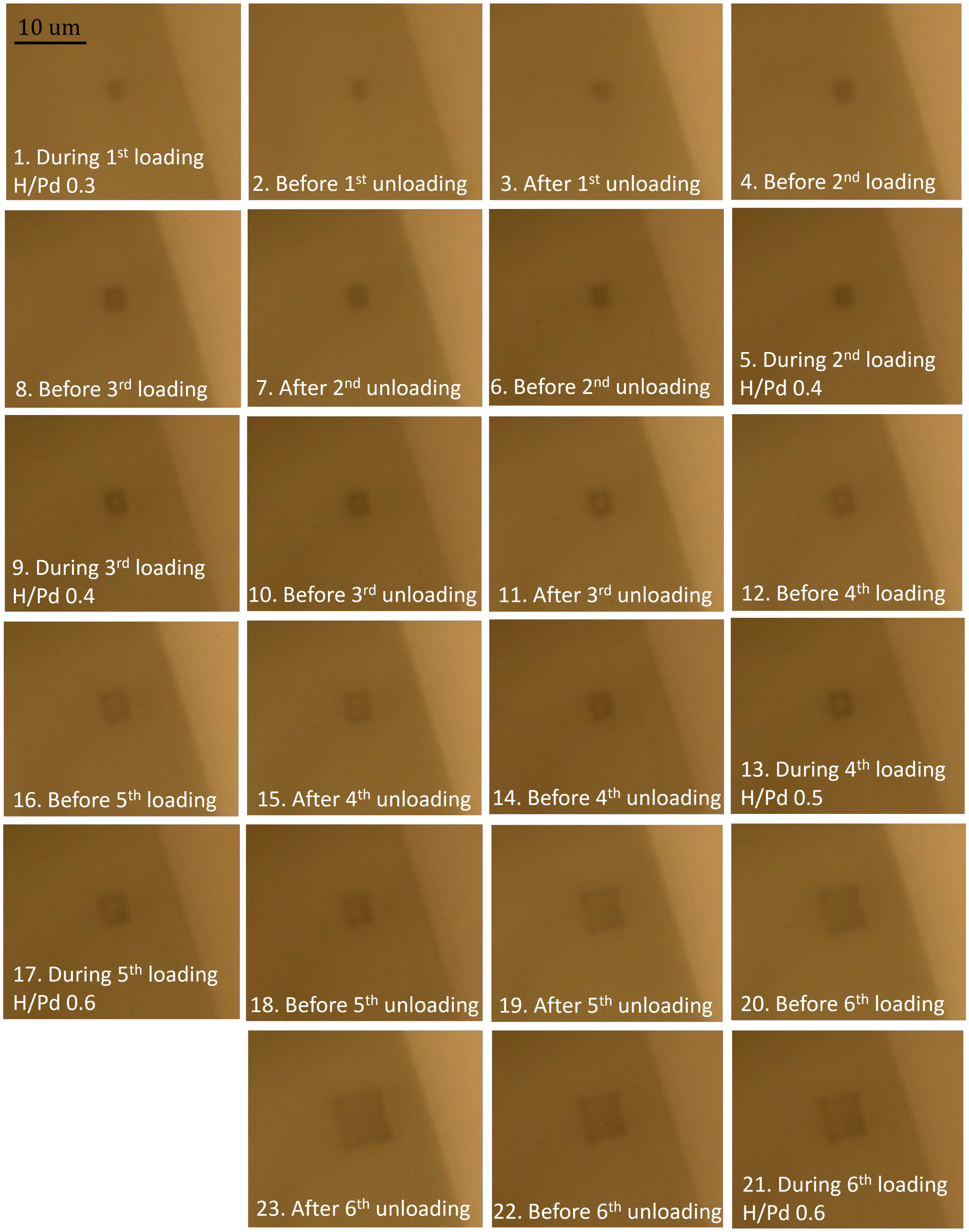

Figure S11: The evolution of a representative defect in the Ge layer as the sample is put through repeated H loading/unloading cycles. As the strain is cycled, the defect starts to grow in size. As can be seen, the defect is oriented along specific crystal axes pointing to the favorable energetics of defect formation for film relaxation under high stress. We empirically observe a higher concentration of defects near the electrode edges where the induced strain is significantly higher.

References

(1) Verma, N.; Delhez, R.; van der Pers, N. M.; Hendrikx, R.; Huizenga, R.; Böttger, A. J. Dislocations, texture and stress development in hydrogen-cycled Pd thin films: An in-situ X-ray diffraction study. *international journal of hydrogen energy* **2022**, *47*, 12119–12134.

(2) Persichetti, L.; Fanfoni, M.; De Seta, M.; Di Gaspare, L.; Ottaviano, L.; Goletti, C.; Sgarlata, A. Formation of extended thermal etch pits on annealed Ge wafers. *Applied Surface Science* **2018**, *462*, 86–94.

(3) Jain, J. R.; Ly-Gagnon, D.-S.; Balram, K. C.; White, J. S.; Brongersma, M. L.; Miller, D. A.; Howe, R. T. Tensile-strained germanium-on-insulator substrate fabrication for silicon-compatible optoelectronics. *Optical Materials Express* **2011**, *1*, 1121–1126.

(4) Nam, D.; Sukhdeo, D.; Roy, A.; Balram, K.; Cheng, S.-L.; Huang, K. C.-Y.; Yuan, Z.; Brongersma, M.; Nishi, Y.; Miller, D.; others Strained germanium thin film membrane on silicon substrate for optoelectronics. *Optics express* **2011**, *19*, 25866–25872.
